# Organizational readiness for implementation of food and nutrition surveillance in Brazilian primary health care: a cross-sectional study

**DOI:** 10.1186/s12913-026-14867-7

**Published:** 2026-07-22

**Authors:** Gabriel Barbosa Delmondes de Moraes, Camila Medeiros da Silva Mazzeti, Rafael Aiello Bomfim, Anderson Leão Nogueira Holsbach, Maria Lígia Rodrigues Macedo, Bruna Paola Murino Rafacho

**Affiliations:** 1 Graduate Program in Health and Development in the Central-West Region, Faculty of Medicine, Federal University of Mato Grosso do Sul, Campo Grande, MS Brazil; 2 Observatório de Condições Crônicas e Alimentação, Faculty of Pharmaceutical Sciences, Food and Nutrition, Federal University of Mato Grosso do Sul, Campo Grande, MS Brazil; 3 Graduate Program in Family Health, Integrated Health Institute, Federal University of Mato Grosso do Sul, Campo Grande, MS Brazil

**Keywords:** Health education, Primary health care, Organizational innovation, Implementation science, Nutrition policy, Health personnel

## Abstract

**Background:**

Food and Nutrition Surveillance is a key strategy of Brazil’s National Food and Nutrition Policy and plays a central role in the organization of nutritional actions in primary health care. Despite its strategic importance, food and nutrition surveillance implementation faces persistent challenges related to training gaps, funding discontinuities, and limited institutional support. This study assessed the organizational readiness of primary health care professionals for food and nutrition surveillance implementation via the Organizational Readiness for Implementing Change (ORIC) instrument.

**Methods:**

This cross-sectional study was conducted in Mato Grosso do Sul, Brazil, during a state-level permanent education event in 2023. Eighty-five primary health care professionals from 49 municipalities responded to the Brazilian version of the ORIC instrument, adapted for the food and nutrition surveillance context. ORIC measures two core constructs: change commitment and change efficacy. The statistical data were analyzed and compared with the readiness levels by professional category (frontline representation) and health macroregion.

**Results:**

The mean ORIC score indicated neutral to moderate readiness across participants (commitment: 3.65; efficacy: 3.71 on a 5-point Likert scale). Nurses and physical education professionals exhibited the highest levels of readiness, surpassing the threshold of 4.0 in both domains. In contrast, nutritionists—despite being the professional group most directly associated with the food and nutrition surveillance—scored lower. Geographic disparities were also evident: professionals from the Eastern Border and Southern Cone regions reported greater readiness than those from the Central and Pantanal macroregions did.

**Conclusions:**

The findings reveal important gaps in organizational readiness for food and nutrition surveillance implementation in primary health care, particularly among nutritionists and in certain territories, suggesting limited engagement or perceived capacity to implement the policy. These results emphasize the need for regionally tailored strategies, stronger integration with permanent education initiatives, and institutional support mechanisms. The ORIC instrument proved to be a useful tool for assessing team-level readiness and guiding implementation efforts. Applying implementation science frameworks may help foster greater alignment between health professionals’ practices and policy goals, ensuring more equitable and sustainable integration of food and nutrition surveillance in primary health care.

**Table:** 

Text box 1. Contributions to the literature
For the first time, this study assesses organizational readiness for the implementation of Food and Nutrition Surveillance (VAN) in Brazilian primary health care via the ORIC instrument; reveals professional and regional disparities, with nurses and physical education professionals showing higher readiness levels than nutritionists do, despite the latter’s central role in VAN; highlights geographic inequalities, with Eastern Border and Southern Cone regions outperforming Central and Pantanal macroregions;
provides actionable insights for tailoring permanent education and institutional support strategies to strengthen VAN implementation; and demonstrates the usefulness of implementation science frameworks, such as ERIC, in guiding targeted interventions in public health systems.

## Background

Food is a social determinant of health and is capable of revealing inequalities in access to resources, influencing nutritional status, and being directly related to the living conditions of the population [1, 2]. Its monitoring occurs through Food and Nutrition Surveillance (Brazilian acronym VAN), a guideline of the Brazilian National Food and Nutrition Policy (Brazilian acronym PNAN), which plays a central role in organizing food and nutrition actions within the Unified Health System. By continuously collecting and analyzing data on the nutritional status and dietary intake of the population served, the VAN supports the planning and evaluation of public policies for health promotion and disease prevention [3, 4].

Operationally, primary health care professionals gather these indicators during routine consultations and input them into national information systems. Local managers and nutritionists then utilize these system reports to identify vulnerable groups, tailor community-specific interventions, and allocate resources [3], thereby strengthening management and guiding interventions according to the principles of universality, equity, and comprehensiveness [5].

In the Brazilian health system, the nutritionist is the professional primarily responsible for leading these actions. Their role encompasses the technical training of primary care teams in anthropometric assessment, providing matrix support to Family Health Strategy units, and managing information systems to guide local nutritional planning [3].

Despite its strategic relevance, VAN implementation in Brazil faces challenges such as shortages of trained human resources, discontinuity of funding, limited systematization of indicators, weak intersectoral coordination, and insufficient recognition of nutritional surveillance as a structuring element of primary health care (PHC), reflecting gaps in training, governance, and technical-institutional support [6].

Addressing these difficulties requires understanding the distinction between adoption and implementation, which are concepts often used interchangeably but with different meanings in implementation science. Adoption refers to the formal introduction of a policy or intervention, whereas implementation involves the concrete effort to put it into practice in real-world settings, with all the inherent variables and challenges [7]. This perspective helps explain why institutionalized policies such as PNAN and VAN do not always produce effective changes in services and care practices. Implementation science offers methods and tools to bridge this gap, analyzing organizational, behavioral, and contextual factors to identify barriers and facilitators that affect the adoption and sustainability of health policies, enabling more realistic and context-sensitive interventions [7].

Among the instruments in this field, the Organizational Readiness for Implementing Change (ORIC) stands out for assessing a team’s or institution’s readiness to implement planned changes. It measures two key dimensions: commitment to change and collective efficacy beliefs regarding implementation [8]. Psychometrically validated in several countries, including Brazil, ORIC has proven to be a practical and useful tool for the Unified Health System services [9]. In the context of VAN, its application can help identify institutional weaknesses, guide strategies for professional development, and strengthen local management, contributing to the consolidation of food and nutrition surveillance in primary health care.

## Methods

### Study aims

This study aimed to determine the level of readiness for the implementation of the VAN among professionals working in primary health care in Brazil across different health macroregions. It was hypothesized that nutritionists would present higher levels of organizational readiness for VAN implementation. While all primary care professionals possess public health training, undergraduate nutrition programs provide specialized instruction on food and nutrition guidelines, knowledge that other professionals often acquire later through in-service experience.

### Study design

This cross-sectional study used the translated Organizational Readiness for Implementing Change instrument (ORIC-Br), which assesses efficacy and commitment in implementing changes in health services. The ORIC-Br is a validated Portuguese-language version of the original ORIC instrument [10]. The version used here [9] was contextually adapted to the VAN setting (e.g., replacing generic terms with specific VAN terminology) without any changes to the original scale’s structure, item wording, or psychometric properties. The instrument, as adapted (Table 2), consists of 12 Likert-scale items (1 = strongly disagree to 5 = strongly agree).

### Study setting and participants

**Fig. 1:**
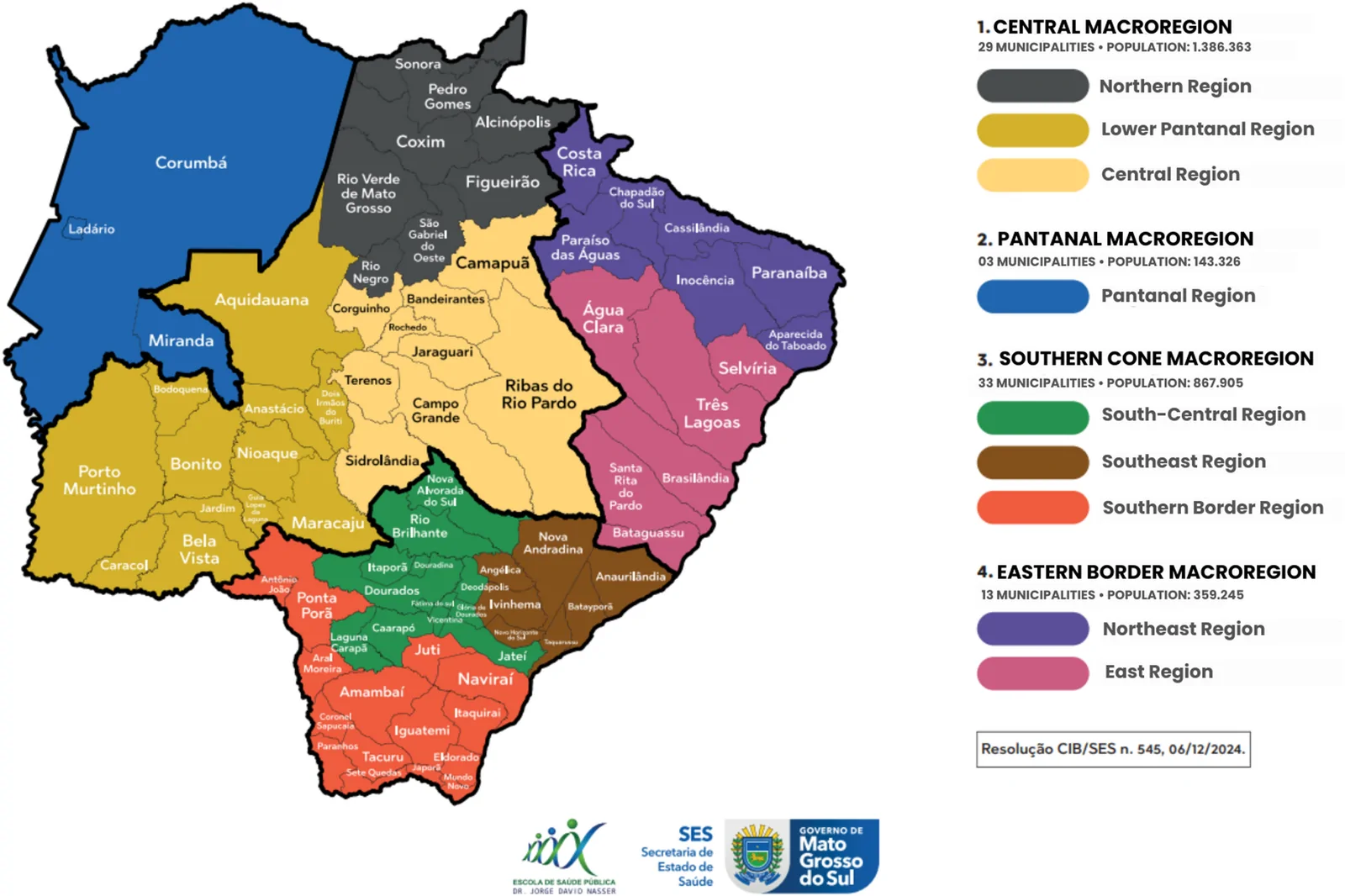
Map of the health macro-regions and regions of the state of Mato Grosso do Sul, Brazil. Source: State Department of Health of Mato Grosso do Sul (SES)

The study was conducted in Mato Grosso do Sul, Brazil, during an in-person continuing education event coordinated by the State Health Department and supported by the *Observatório de Condições Crônicas e Alimentação* in September 2023. Eighty-five frontline primary health care professionals involved in the VAN process [11] voluntarily completed a SurveyMonkey questionnaire [12]. Rather than holding managerial roles, participants were designated by their municipalities as local technical references for VAN. Because data collection occurred during a state-level event in the capital (Campo Grande), attendance relied strictly on municipal approval, scheduling availability, and travel funding. Mato Grosso do Sul is divided into four health macroregions; for a comprehensive understanding of this division, Fig. 1 displays this information below.

The study aimed to identify the highest mean readiness score within professional groups regarding the implementation of VAN in their work dynamics.

### Data collection

The questionnaires were completed individually in a controlled, quiet environment. Anonymity, confidentiality, and physical distancing were ensured. Standardized instructions were read by a trained facilitator external to the participants’ teams.

### Data analysis

The data were analyzed via measures of central tendency and dispersion. The covariates included municipality, professional background, and position in primary health care. Mean Likert scores were examined globally and by education, with geographic variables used to assess associations [13]. Analyses were performed in Stata 17.0 [14] and Jamovi 2.7.24 [15], applying the Shapiro–Wilk test. The non-parametric Kruskal-Wallis test was used to compare readiness scores across professional categories. ORIC scores were calculated per item and domain. The > 4 threshold was used as a descriptive benchmark of high readiness, consistent with prior ORIC-Br applications [9]. Because the domain scores were not normally distributed, inferential comparisons were based on continuous scores using non-parametric tests rather than on categorical cut-offs. The study followed the STROBE guidelines [16, 17]. To address potential sampling biases and ensure statistical reliability, professional categories with very small sample sizes (e.g., social workers and physiotherapists) were grouped into a single category labeled ‘Other primary health care Professionals’ for subgroup analysis.

### Ethics approval and consent to participate

This study was approved by the Research Ethics Committee at the Federal University of Mato Grosso do Sul (CAAE: 66044322.9.0000.0021). Written informed consent was obtained from all individuals who chose to participate in the study.

## Results

As shown in Table 1, a total of 49 municipalities responded (62% of Mato Grosso do Sul’s municipalities), yielding 85 valid responses.

**Table 1:** Professional category of participants

Professional Background	*n*	%
Physical Education	5	5.9
Nursing	11	12.9
Nutrition	53	62.4
Psychology	8	9.4
Other multidisciplinary professionals*	8	9.4
Total	85	100

*The category ‘Other multidisciplinary professionals’ is composed of Physioterapists (n = 7) and Social Worker (n = 1)

The mean values ranged between 3 and 4 points (3.17 for Commitment and 3.65 for Efficacy), which, according to the Likert scale, may indicate a neutral stance regarding the motivation to implement the VAN, as shown in Table 2.

In the second analysis, the average scores by professional background showed that physical education professionals (4.27 – Commitment, and 4.06 – Efficacy) and nurses (4.12 – Commitment, and 4.0 – Efficacy) were the most willing to implement VAN. Nutritionists, although trained to work with VAN, presented lower scores (3.67 – Commitment, and 3.70 – Efficacy), indicating a neutral stance regarding its implementation in primary health care. A Kruskal-Wallis test revealed no statistically significant differences between professional categories regarding organizational readiness for both efficacy (*p* = 0.572) and commitment (*p* = 0.418), as described in Table 3.

**Table 2:** Individual analysis of the items and average score by questionnaire domain

Item	*n*	Mean ± SD	Median (P25-P75)	Readiness ( > = 4 Likert scale)
People working here (at the health unit or e-MULTI) feel confident that the organization of all involved actors will enable people to engage in the implementation of this change (execution of food and nutrition surveillance).	85	3.71±0.1.26	4 (3–5)	59 (69.4%)
People working here (at the health unit or e-MULTI) are committed to implementing this change (execution of food and nutrition surveillance in PHC).	85	3.73 ± 1.2	4 (3–5)	58 (68.2%)
People working here (at the health unit or e-MULTI) feel confident that they will be able to monitor the progress of implementing this change (execution of food and nutrition surveillance in PHC).	85	3.62 ± 1.3	4 (3–5)	54 (63.53%)
People working here (at the health unit or e-MULTI) will do whatever is necessary to implement this change (execution of food and nutrition surveillance in PHC).	85	3.64 ± 1,16	4 (3–5)	51 (60.0%)
People working here (at the health unit or e-MULTI) believe that the organization (Health Department) will support them as they adapt to the change (execution of food and nutrition surveillance in PHC).	85	3.78 ± 1.14	4 (3–5)	54 (63.5%)
People working here (at the health unit or e-MULTI) want to implement this change (execution of food and nutrition surveillance in PHC).	85	3.5 ± 1.09	4 (3–5)	51 (60.0%)
People working here (at the health unit or e-MULTI) feel confident they will be able to maintain the pace of implementation of this change (execution of food and nutrition surveillance in PHC).	85	3.64 ± 1.13	4 (3–5)	52 (61.2%)
People working here (at the health unit or e-MULTI) feel confident they will be able to overcome the challenges that may arise during implementation of this change (execution of food and nutrition surveillance in PHC).	85	3.75 ± 1,25	4 (3–5)	58 (68.2%)
People working here (at the health unit or e-MULTI) are determined to implement this change (execution of food and nutrition surveillance in PHC).	85	3.85 ± 1.32	4 (3–5)	60 (70.5%)
People working here (at the health unit or e-MULTI) are motivated to implement this change (execution of food and nutrition surveillance in PHC).	85	3.68 ± 1.24	4 (3–5)	62 (72.9%)
People working here (at the health unit or e-MULTI) feel confident they will be able to manage the implementation policy for this change (execution of food and nutrition surveillance in PHC).	85	3.55 ± 1.28	4 (3–5)	54 (63.5%)
Efficacy domain	85	3.71 ± 1.10	4 (3−4.6)	44 (51.76%)
Commitment domain	85	3.65 ± 0,99	3.8 (3,1–4,8)	41 (48.24%)
Overall average score	85	3.68 ± 1.02	3.8 (3.1–4.4)	37 (43.53%)

PHC: primary care health; *n* = 85 respondents

**Table 3:** Mean scores for efficacy and commitment by professional category

Professional background	*n*	Mean Commitment ± SD	*n* Ready (> 4 Commitment)	Mean Efficacy ± SD	*n* Ready (> 4 Efficacy)
Overall	85	3.7 1 ± 1.1	41(48.24%)	3.6 5 ± 0.99	44 (51.7%)*
Physical Education	5	4.24 ± 0.6	3 (60.0)*	4.06 ± 0.81	4 (80.0)*
Nursing	11	4.12 ± 0.93	7 (63.6)*	4.0 ± 0.98	7 (63.6)*
Nutrition	53	3.67 ± 0.82	24 (45.2)	3.70 ± 1	27 (50.9)*
Psychology	8	3.42 ± 1.5	4 (50.0)*	3.31 ± 1.48	3 (37.5)
Other multidisciplinary professionals**	8	3,13 ± 1.55	3 (37,5)	3.17 ± 1.49	3 (37,5)

*Values above 50% indicate favorable readiness for VAN implementation

**The category ‘Other multidisciplinary professionals’ is composed of Physioterapists (n = 7) and Social Worker (n = 1). Non-parametric data was analysed using the Kruskal-Wallis test

Table 4 details the organizational readiness scores distributed across the state’s health macroregions. The Southern Cone and Central macroregions accounted for the majority of the sample (*n* = 40 and *n* = 31, respectively). Professionals from the Eastern Border reported the highest mean scores for both efficacy (3.98 ± 1.04) and commitment (3.95 ± 0.85), culminating in the highest overall readiness proportion (66.7%). Conversely, the Central and Pantanal macroregions exhibited the lowest average scores across all domains. It is important to note the extremely small number of respondents from the Pantanal (*n* = 2);

**Table 4:** Mean scores for efficacy and commitment by state health macroregion

Macroregion	Efficacy Mean ± SD	Efficacy Readiness% above 4 points	Commitment Mean ± SD	Commitment Readiness % above 4 points	Total Mean ± SD	Overall Readiness
Central *n* = 31	3.46 ± 1,14	12 (38.71%)	3.45 ± 1.06	13 (41.94)	3.46 ± 1.08	10 (32.3)
Southern Cone *n* = 40	3.84 ± 1.08	23 (57.5%)*	3,72 ± 0.92	20 (50.0%)*	3.77 ± 0.99	18 (45.0)
Eastern Border *n* = 12	3.98 ± 1.04	8 (66.7%)*	3.95 ± 0.85	7 (58.3)*	3.96 ± 0.90	8 (66.7)*
Pantanal *n* = 2	3.4 ± 1.13	1 (50.0%)*	3.5 ± 1.41	1 (50.0)*	3.45 ± 1.28	1 (50.0)*

*Values above 50% indicate favorable readiness for VAN implementation

**Percentage of individuals with scores above 4 points

## Discussion

Considering the historical challenges of permanent education and the central role of VAN in structuring food public policies, this analysis of organizational readiness for VAN implementation in primary health care reveals both progress and weaknesses in the coordination between professionals, management, and health guidelines.

The participants represented 49 of the 79 municipalities in Mato Grosso do Sul. The analysis of the commitment and efficacy domains revealed disparities, as summarized in Table 4, in which the Eastern Border had the highest efficacy (66.7%), followed by the Southern Cone (57.5%), and the Central and Pantanal regions had lower percentages (38.7% and 50.0%). For commitment, the Eastern Border led (58.3%), whereas Central and Pantanal showed lower levels of engagement (41.9% and 50.0%, respectively).

With a cutoff of 4 on the Likert scale and 50% agreement as the threshold [18], only nursing, physical education, and specific macroregions (Eastern Border, Southern Cone) presented favorable conditions. Other groups, such as nutritionists and the Central macroregion, indicated neutrality or low readiness. These results call for differentiated, regionally adapted strategies to strengthen EPS, enhance VAN recognition, and ensure institutional support.

A comparison with the literature confirms that Eastern Border and Southern Cone—areas with better infrastructure, managerial support, and lower interregional dependency—align with positive determinants for VAN [6, 19]. Conversely, the Central and Pantanal macroregions’ lower scores may reflect infrastructure gaps, urban overload, and reliance on external support [20, 21]. Strategic human resource management studies reinforce that integrated management and EPS correlate with greater commitment [22].

The demand for integrated management and robust training is not exclusive to the Brazilian scenario, when compared to global primary health care settings. In countries like the UK, Canada, and Australia, the integration of nutritional care often falls to general practitioners or nurses, who frequently report a lack of specialized training, time constraints, and competing clinical priorities as major hurdles to effective nutritional surveillance [23, 24]. While the Brazilian model offers a unique structural advantage by incorporating nutritionists directly into the primary health care matrix support teams, our findings demonstrate that without continuous local management support and adequate infrastructure, the mere presence of specialized professionals is insufficient to overcome systemic operational barriers. To truly activate the potential of these teams and translate their specialized knowledge into effective surveillance, continuous institutional support and structured educational policies are essential.

The National Policy for Permanent Education in Health (2004) tasks the Ministry of Health with supporting managers in planning and implementing quality improvement actions [25, 26]. EPSs strengthen the Unified Health System by promoting critical, reflective training based on service needs [27] and fostering integration, problem-solving, and humanized care [28]. However, municipal support is inconsistent with funding, infrastructure, and trained facilitator shortages [29]. The proposed solutions include municipal EPS centers aligned with health plans and supported by teaching-service integration committees [30], as well as stronger state/federal technical support [26]. Sustaining EPS requires political, institutional, and operational commitments.

Implementation science offers a lens to translate EPS evidence into practice, stressing adaptation with stakeholders and contextual tailoring [31]. For VAN, barriers such as low readiness among nutritionists, regional disparities, and institutional neutrality require structured strategies. To bridge the gap between assessment and action, it’s suggested to apply tailored strategies from the Expert Recommendations for Implementing Change (ERIC) compilation, a validated taxonomy that provides a standardized menu of implementation strategies designed to address specific contextual barriers [32]. Specifically, the neutral commitment observed could be addressed by engaging local leaders and using local data to demonstrate the practical value of VAN. Furthermore, interactive educational interventions and continuous supervision can help overcome the reported training gaps and better prepare key professionals. However, challenges remain in mobilizing primary health care professionals.

However, challenges remain in mobilizing primary health care professionals. When the ORIC-Br items were individually assessed, the scores appeared to be homogeneous across the questionnaire domains, generally reflecting a neutral stance. This neutrality reflects operational gaps and possible resistance to established policies. Historically, the Brazilian public health movement has challenged the traditional biomedical model by treating health as a fundamental social right rather than merely a clinical issue. This perspective links healthcare reform directly to broader social equity. However, the adoption of new daily practices remains limited by entrenched cultural and ideological barriers [33].

Professionals face heavy workloads and shortages, making neutrality a pragmatic choice that is reinforced by insufficient institutional support [34]. Many lack training in advocacy and policymaking, reducing confidence in engaging politically [35]. Feeling undervalued and excluded from decisions fosters disengagement [36]. Unclear communication about reforms can heighten insecurity and resistance.

It is precisely within this broader context of systemic exhaustion that the specific findings concerning nutritionists must be understood. The apparent contradiction regarding the lower perceived readiness among nutritionists warrants careful interpretation. It is crucial to note that VAN is not a future initiative, but an ongoing, mandatory routine within the SUS. Given their firsthand knowledge of the complexities of food and nutrition policies, nutritionists already experience chronic structural deficits, such as inadequate equipment and high demands. Therefore, quantitative tools like ORIC might capture this pragmatic realism and ‘change fatigue’ as lower organizational readinessIndeed, their lower scores may reflect accumulated exposure to implementation barriers and a more critical appraisal of the feasibility of VAN in routine practice, rather than lower commitment per se. What appears as a lack of readiness may, in fact, be an acute awareness of the systemic conditions necessary to implement effective nutritional care, which are often unmet.

Nurses’ effectiveness in this study may be related to leadership roles, communication skills, and crisis adaptability, as observed during the COVID-19 pandemic [37]. Physical education professionals also demonstrated high readiness, which is likely tied to their strategic role within the Health Academy Program [38, 39]. As a structural component of the Primary Health Care network, this health promotion strategy operates through publicly funded hubs equipped with adequate infrastructure and qualified staff to deliver guided physical activities and care directly to the community. Due to their high readiness scores and ability to actively support VAN data collection, both groups emerge as key target audiences for leadership roles in primary care [40].

Neutrality among nutritionists and low readiness in the central region risk widening inequalities. Equity requires technical support, training, and proportional resources. PNAN’s limited dissemination among nutritionists reduces its impact [3]. Gaps in integrating policies into curricula and practice hinder understanding and application [41, 42]. The absence of monitoring and feedback mechanisms, coupled with local coordination challenges, further limits engagement [41–43].

## Strengths and limitations

Regarding the study’s limitations, it is important to consider the context in which participants are embedded. Some respondents reported limited municipal support for training, which seems to affect their motivation to implement food and nutrition surveillance in routine practice. This may, in turn, have influenced the readiness scores observed. In addition, the lack of more detailed sociodemographic information, such as age, years of professional experience, and time in the current position; restricted a more thorough analysis of potential confounders that could help explain variations in readiness. It is also worth noting that the sample was largely composed of nutritionists, and that data were collected in group settings, which may have introduced both selection and response biases.

Despite these challenges, ORIC’s strengths include psychometric robustness, practicality, and the ability to capture team-level readiness, providing strategic insights. Its specific limitations include a lack of comparative studies, the absence of a test–retest reliability, and sensitivity to contextual factors in unequal regions. As a quantitative tool, ORIC may miss organizational complexities; so complementing it with qualitative methods is recommended.

## Conclusion

This study assessed organizational readiness for the implementation of the Food and Nutrition Surveillance System (VAN) within the scope of primary health care in Brazil, using the ORIC instrument to capture health professionals’ perceptions regarding commitment to and efficacy of change. Contrary to the initial hypothesis, nutritionists did not show the highest levels of readiness; instead, nurses and physical education professionals demonstrated greater perceived preparedness for implementation. These findings suggest the existence of structural and educational gaps that may limit the leadership role of nutritionists in operationalizing food and nutrition public policies at the local level.

The results highlight the importance of institutional support, integration with permanent education in health initiatives, and contextual factors in shaping organizational readiness. The study also reinforces the practical value of the ORIC instrument in identifying organizational facilitators and barriers, thereby informing targeted strategies to strengthen the capacity for change in public health systems.

To advance the implementation of VAN, sustained and coordinated efforts among different professional categories are essential, along with curricular improvements and stronger institutional support. The adoption of strategies such as reinforcing the commitment of local leaders, preparing key professionals to lead the process, offering continuous supervision, using local data to justify change, and promoting interactive educational interventions—as recommended by the ERIC framework—can support the integration of policy guidelines into service routines, ensuring that they are not only formally adopted but also effectively incorporated into practice.

## Data Availability

The datasets used and/or analyzed during the current study are available from the corresponding author upon reasonable request.

## Abbreviations

VAN: Food and Nutrition Surveillance
PNAN: Brazilian National Food and Nutrition Policy
PHC: Primary Health Care
ORIC: Organizational Readiness for Implementing Change
EPS: Permanent Health Education
